# Pharmacological mechanism and clinical application of ciprofol

**DOI:** 10.3389/fphar.2025.1572112

**Published:** 2025-03-25

**Authors:** Jianshun Zhou, Lifeng Wang, Zhaoying Zhong, Lei Yuan, Jinhua Huang, Ping Zou, Xiaohui Cao, Donglan Peng, Baozhen Liao, Jianqiang Zeng

**Affiliations:** ^1^ Department of Anesthesiology, Ganzhou Cancer Hospital, Ganzhou, Jiangxi, China; ^2^ Department of Anesthesiology, First Affiliated Hospital of Gannan Medical University, Ganzhou, Jiangxi, China; ^3^ Ganzhou Key Laboratory of Anesthesiology, The First Affiliated Hospital of Gannan Medical University, Ganzhou, Jiangxi, China

**Keywords:** propofol, ciprofol, GABA, anesthesia, sedation

## Abstract

Propofol has become one of the most commonly used anesthetic agents because of its good sedative effects, rapid onset, and fast metabolism. However, its associated respiratory and circulatory depression and injection pain make it difficult for patients to tolerate. Ciprofol, which is structurally similar to propofol but has an additional cyclopropyl group, is less likely to impact respiratory and circulatory function and cause injection pain, highlighting its potential for clinical application. Currently, as research on Ciprofol is still in the exploratory stage, its clinical application is limited because its underlying mechanisms are not yet fully understood. The aim of this article is to review the pharmacological mechanisms of propofol, hypothesize the primary pharmacological effects and potential adverse reactions of Ciprofol, and summarize its current clinical application status, with the goal of providing a reference for future clinical use.

## 1 Pharmacological characteristics of ciprofol

**FIGURE 1:**
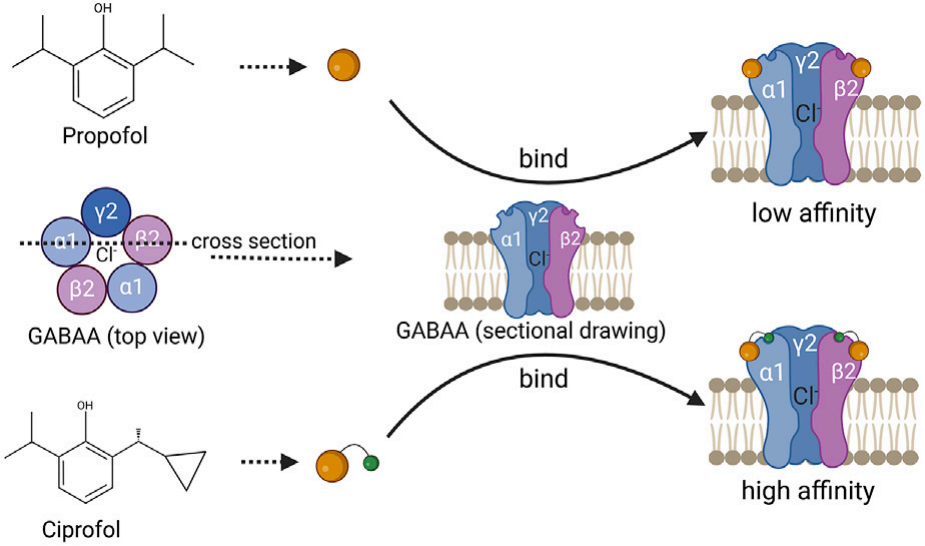
Schematic diagram of the chemical structure of propofol and ciprofol, with a schematic diagram of their binding to GABAA. The above is a schematic diagram of the chemical structure of propofol, and the following is a schematic diagram of the chemical structure of ciprofol, which binds more closely to the GABAA receptor and has a higher affinity.

Ciprofol is a novel GABA-A receptor agonist that is structurally similar to propofol but with the addition of a cyclopropyl group and exists as both R- and S-enantiomers (Qin et al., 2017) (Figure 1). Studies suggest that the incorporation of the R-chiral center and cyclopropyl group causes a structural change that provides a better spatial volume, moderately increasing its lipophilicity and pharmacological properties. As a result, ciprofol may bind to the GABA-A receptor more tightly than propofol does, causing an anesthetic potency approximately four to five times greater than that of propofol (Qin et al., 2017; Hu et al., 2021; Lu et al., 2023; Luo et al., 2022). Moreover, owing to its increased lipophilicity, ciprofol crosses the blood-brain barrier, demonstrating wide and rapid distribution in tissue. This may account for its rapid onset of central nervous system effects (Hung et al., 2023). It has been reported that a single intravenous dose of 0.4 mg kg^−1^ ciprofol can induce hypnosis within 2 min, with an average time to awakening of 10.6 min and a minimum of 5.5 min (Bian et al., 2021).

The primary metabolic pathways of ciprofol include oxidation, glucuronidation, and sulfation, with the major metabolite in plasma being the glucuronide conjugate M4 (79.3%), which is pharmacologically inactive and primarily excreted via the kidneys (Bian et al., 2021). Compared with propofol, ciprofol exerts minimal effects on the respiratory and circulatory systems and is less likely to cause injection pain (Li J. et al., 2022; Wu et al., 2022; Zhu et al., 2023). Therefore, ciprofol appears to have promising clinical applications, and understanding its main pharmacological effects and mechanisms of adverse reactions is crucial for its safe clinical use.

## 2 Potential sedative mechanism

γ-Aminobutyric acid (GABA) is the major inhibitory neurotransmitter that controls synaptic transmission and neuronal excitability in the central nervous system (CNS), is widely distributed in the brain, and maintains a balance between excitation and inhibition in the cerebral cortex (Kim and Hibbs, 2021). GABA has three receptor subtypes, namely, GABA_A_, GABA_B_, and GABA_C_ (Shimizu-Okabe et al., 2022). Among these, GABA_A_ is a ligand-gated ion channel composed of α1-6, β1-3, γ1-3, δ, ε, Φ, and π subunits that can form different subtypes. The most common subtype found in the brain is α1β2γ2, which is also the primary receptor targeted by propofol (Kim and Hibbs, 2021; Solomon et al., 2019; Kim et al., 2020).

Ciprofol, a novel γ-aminobutyric acid (GABA) receptor inhibitor, shares a similar chemical structure with propofol and exerts its anesthetic and sedative effects primarily through the activation of GABA_A_ receptors (Solomon et al., 2019; Duan et al., 2023). Upon activation of GABA_A_ receptors by ciprofol, chloride ions influx, and ciprofol also competes with tert-butylbicyclophosphorothionate (TBOB) and tert-butylbicyclophosphorothionate (TBPS) for competitive binding to the chloride ion channel, enhancing the chloride influx induced by GABA, leading to membrane hyperpolarization and inhibiting the transmission of excitatory neurotransmitters (such as glutamate) between neurons, thereby suppressing the central nervous system and producing sedative effects (Kim et al., 2020; Liao et al., 2022) (Figure 1).

**FIGURE 2:**
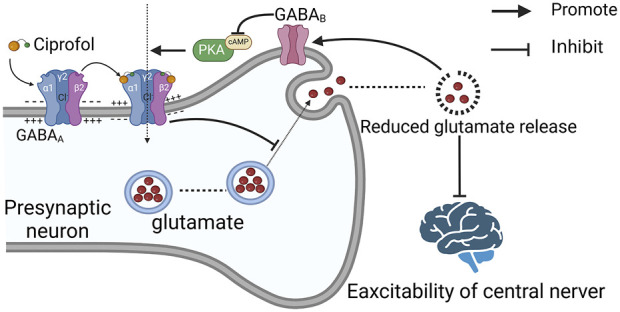
Schematic diagram of the mechanism of the central sedative action of ciprofol production. The binding of ciprofol to the GABAA receptor results in a change in membrane potential, inhibiting glutamate release and ultimately producing a sedative effect. AMP: cyclic adenosine monophosphate, PKA: cyclic AMP-dependent protein kinase.

Furthermore, studies have shown that glutamate can reduce the expression and activation of GABA_B_ through the NMDA receptor (Guetg et al., 2010; Xu et al., 2014; Maier et al., 2010). Therefore, low concentrations of glutamate may promote the activation of GABA_B_ receptors. The GABA_B_ receptor is a seven-transmembrane G protein-coupled receptor that, once activated, inhibits adenylyl cyclase, reducing cyclic adenosine monophosphate (cAMP) synthesis and leading to a decrease in the cAMP concentration (Li et al., 2020). As cAMP is an important neurotransmitter for activating protein kinase A, a decrease in the cAMP concentration reduces the activity of the cAMP‒PKA signaling pathway, further inhibiting neuronal excitability and synaptic transmission and enhancing the sedative effect (Connelly et al., 2013) (Figure 2). The activation of GABA_B_ may also enhance the sustained GABA_A_ current through downstream regulation by PKA, further strengthening central inhibition and achieving rapid sedation. Currently, some studies suggest that the anesthetic effects of propofol may be attributed to the activation of GABAB receptors (Schwieler et al., 2003); however, direct evidence regarding the relationship between ciprofol and GABAB receptors is still lacking. Nevertheless, this may provide a new avenue for research into the mechanism underlying the stronger anesthetic potency of ciprofol: in addition to differing affinities for GABA receptors, it may also be related to selective differences in binding to the GABAA and GABAB subtypes.

## 3 Mechanisms of potential adverse reactions

### 3.1 Cardiovascular depression

One of the common adverse reactions of ciprofol is hypotension, which, similar to propofol, may involve multiple mechanisms. Studies have revealed that GABA can regulate renal sympathetic and visceral sympathetic nerve activity through GABA_A_ receptors, leading to changes in cardiovascular activity (Milanez et al., 2020). Therefore, it is likely that ciprofol, like propofol, induces hyperpolarization of neuronal membranes upon activation of GABA_A_ receptors, which in turn inhibits renal and visceral sympathetic nerve activity, resulting in a decrease in blood pressure (Ebert et al., 1992). Additionally, ciprofol may cause blood pressure reduction by indirectly dilating blood vessels through other mechanisms, possibly involving a protein kinase C (PKC)-dependent pathway (Noguchi et al., 2023).

**FIGURE 3:**
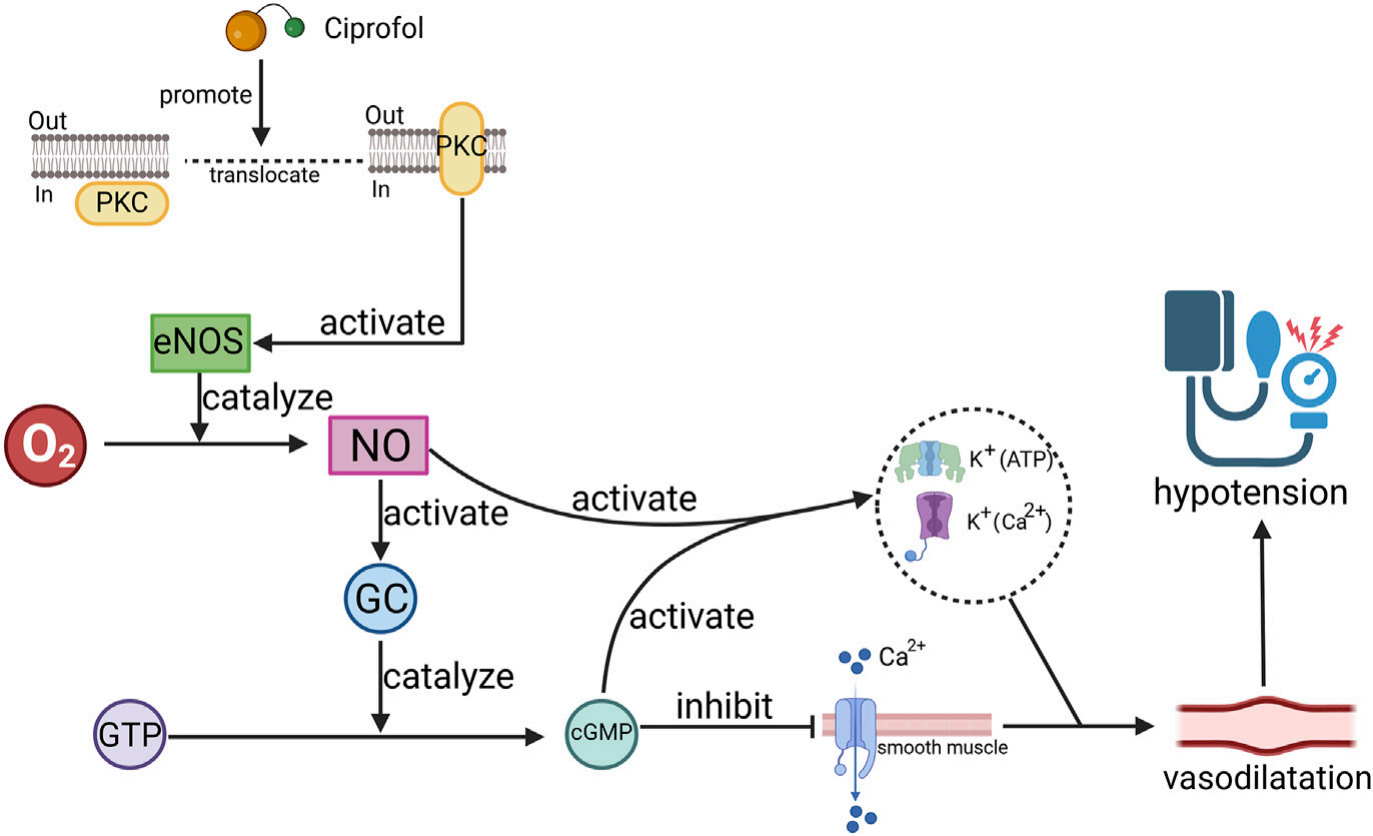
Schematic diagram of the mechanism by which ciprofol causes hypotension. Ciprofol promotes the release of nitric oxide (NO) by promoting protein kinase C (PKC) translocation, activating endothelial nitric oxide synthase (eNOS), thereby causing cyclic guanosine monophosphate (cGMP), which ultimately leads to vascular smooth muscle relaxation. O_2_: oxygen, GC: guanylate cyclase, GTP: guanosine triphosphate, K^+^(Ca^2+^): calcium-activated potassium channels, K^+^ (ATP): ATP-sensitive potassium channels.

Researchers have reported that propofol can stimulate the translocation of PKC isoforms, leading to the activation of endothelial nitric oxide synthase (eNOS) (Wang et al., 2010), which increases nitric oxide (NO) levels. The generated NO activates guanylate cyclase (GC), increasing cyclic guanosine monophosphate (cGMP) production. This, in turn, reduces calcium influx into smooth muscle cells, resulting in smooth muscle relaxation and vasodilation (Nagakawa et al., 2003; Kamitani et al., 1995; Yamanoue et al., 1994). Moreover, the activation of NO and cGMP can lead to the activation of calcium-activated potassium channels (K^+^ (Ca^2+^)) and ATP-sensitive potassium channels (K^+^ (ATP)), inducing hyperpolarization and relaxation of vascular smooth muscle and further promoting vasodilation (Nagakawa et al., 2003) (Figure 3). eNOS activation also decreases intracellular calcium concentrations and reduces myocardial sensitivity to calcium, thereby inhibiting myocardial contractility and contributing to blood pressure reduction (Kanaya et al., 2005; Wickley et al., 2006). Additionally, propofol may lower the calcium threshold for the activation of arterial smooth muscle cell channels by increasing the calcium sensitivity of large conductance calcium-activated potassium channels (BKCa), resulting in greater vasodilation (Liu et al., 2012). Notably, propofol may also reduce the production of cAMP through PKC activation, thereby diminishing β-adrenergic signaling in cardiomyocytes and reducing the heart’s excitatory response to β-adrenergic receptor activation (Kurokawa et al., 2002). Recent studies have revealed that ciprofol can attenuate myocardial injury caused by β-agonists, suggesting that it may exert similar effects on β-receptors (Yang et al., 2022).

Unfortunately, research on the mechanisms underlying ciprofol-induced hypotension is limited. Given the structural and pharmacological similarities between ciprofol and propofol, we speculate that their mechanisms of action may be similar. However, in clinical practice, ciprofol appears to have a milder effect on the cardiovascular system. We hypothesize that this may be due to the following reasons: 1. Ciprofol is a more potent anesthetic, thus the doses administered in clinical settings are typically lower than those of propofol, resulting in lower plasma concentrations and reduced effects on receptors or channels involved in cardiovascular depression. 2. Ciprofol is metabolized more rapidly at lower doses, and its metabolites are pharmacologically inactive, leading to a shorter duration of cardiovascular suppression than that of propofol (Bian et al., 2021).

### 3.2 Respiratory depression

Both ciprofol and propofol inevitably cause respiratory depression, which is closely related to their action on GABA receptors. The preBötzinger complex (preBötC) in the ventral medulla of mammals regulates normal respiratory rhythm (Song et al., 2016; Kam et al., 2013), and the role of the neurotransmitter GABA in this process is significant (Johnson et al., 2002; Ghali and Beshay, 2019). Studies have revealed that high concentrations of GABA in the ventricular pool can significantly reduce tidal volume in dogs (Kazemi and Hoop, 1991). This regulation is likely mediated through the activation of GABA_A_ receptors, as research has indicated that GABA_A_ receptor antagonists in the brainstem significantly increase the frequency and amplitude of respiratory-modulated hypoglossal (XII) neuron bursts, enhancing the control of respiratory rhythm (Johnson et al., 2002).

As an agonist of GABA_A_ receptors, ciprofol is likely to inhibit preBötC’s regulation of respiratory rhythm, leading to respiratory depression. Additionally, studies have revealed that GABA_B_ receptor agonists can cause significant respiratory depression (Follman and Morris, 2022), although whether GABA_B_ receptors are involved in ciprofol-induced respiratory depression requires further investigation. Interestingly, despite the structural similarity between ciprofol and propofol, ciprofol tends to cause less severe and less frequent respiratory depression (Lan et al., 2023). This difference may be related to their distinct interactions with GABA_A_ receptor subunits. Propofol has a relatively high affinity for the β2 and β3 subunits (Jonsson Fagerlund et al., 2010; Jonsson Fagerlund et al., 2012), and its sedative effects are primarily mediated by these subunits (Zeller et al., 2005). Respiratory depression induced by propofol is mainly mediated by the β3 subunit (Zeller et al., 2005), suggesting that ciprofol may predominantly target different subunits, thereby resulting in milder respiratory depression.

Moreover, since ciprofol is four to five times more potent than propofol is, the doses administered in clinical settings are lower, leading to lower plasma concentrations, faster metabolism, and a shorter duration of action. Compared with that of propofol, its main metabolite, M4, does not have any toxic or hypnotic properties (Qin et al., 2017; Bian et al., 2021), which further contributes to its shorter duration and milder manifestation of respiratory depression.

### 3.3 Injection pain

Injection pain is a common clinical issue associated with propofol, as evidenced by an incidence as high as 60% (Jalota et al., 2011). This is thought to be caused by direct stimulation of the free afferent nerve endings between the tunica media and intima of the vein or by the activation of the kinin cascade, resulting in the release of mediators such as kininogen when the active ingredients in the propofol emulsion come into contact with the vascular endothelium (Tan and Onsiong, 1998). Additionally, the intensity of injection pain is proportional to the concentration of the drug in the aqueous phase of the emulsion (Jalota et al., 2011). Although ciprofol has a chemical structure similar to that of propofol and a similar mechanism for causing injection pain, the incidence of injection pain is significantly lower (Hu et al., 2021; Luo et al., 2022), possibly because of multiple factors. First, ciprofol is a more potent anesthetic than propofol is, so the doses administered in clinical settings are typically lower (Qin et al., 2017; Klement and Arndt, 1991). Second, ciprofol, which has an additional cyclopropyl group, has significantly increased lipophilicity and hydrophobicity (Qin et al., 2017). As a result, it is typically formulated as a water-in-oil emulsion, which further reduces its water solubility. In emulsions with the same lipid concentration, the aqueous phase concentration of ciprofol is significantly lower than that of propofol (Qin et al., 2017). Additionally, the higher lipophilicity of ciprofol allows it to rapidly cross cell membranes and distribute into other tissues (Hung et al., 2023), further lowering the concentration of free drug in the aqueous phase. The concentration of free drug in the aqueous phase is a key factor in determining whether injection pain occurs, and since the aqueous phase concentration of ciprofol is lower than that of propofol, the likelihood of injection pain is correspondingly reduced (Ding et al., 2022).

## 4 Clinical applications

### 4.1 General anesthesia induction and maintenance

#### 4.1.1 Anesthesia induction

Ciprofol is being increasingly incorporated into clinical practice, and its safety and efficacy are being more frequently validated. For anesthesia induction, 0.3–0.5 mg kg^−1^ ciprofol is not inferior to 2.0–2.5 mg kg^−1^ propofol (Wang et al., 2022). A single infusion of 0.4 mg kg^−1^ ciprofol is pharmacokinetically stable and safely and successfully induces anesthesia in healthy individuals (Bian et al., 2021; Wang et al., 2022). In clinical studies, researchers reported that a dose of 0.4 mg kg^−1^ ciprofol successfully induced anesthesia in 100% of patients and was associated with a low incidence of poor responses to intubation and a significantly smaller drop in blood pressure within the first 10 min after induction than 2.0 mg kg^−1^ propofol was (Chen et al., 2022). Owing to individual differences, 0.3 mg kg^−1^ is more suitable for elderly patients (aged ≥65), with similar efficacy to 0.4 mg kg^−1^ in younger patients, maintaining stable hemodynamics and a low incidence of respiratory depression (Duan et al., 2023; Li et al., 2021). Notably, the incidence of hypotension caused by the same dose of ciprofol increases with age (Lu et al., 2024). In contrast to elderly patients, children require a higher induction dose. A study revealed that a dose of 0.6 mg kg^−1^ in children results in stable circulation and BIS values (Pei et al., 2023).

Ciprofol has been successfully used in various noncardiac surgeries, including abdominal, orthopedic, urological, thoracic, and neurosurgical procedures, and has demonstrated favorable outcomes (Ding et al., 2022).

#### 4.1.2 Anesthesia maintenance

For anesthesia maintenance, ciprofol at a rate of 0.9 mg kg^−1^ h^−1^ provides similar anesthetic potency to isoflurane at 5.7 mg kg^−1^ h^−1^ (Liang et al., 2023). A population pharmacokinetic-pharmacodynamic (PK-PD) model suggests that the optimal maintenance dose of ciprofol is 0.8 mg kg^−1^ h^−1^ during surgery (Liu L. et al., 2024). A phase III clinical trial revealed that after induction with 0.4 mg kg^−1^ ciprofol, anesthesia was maintained at an initial rate of 0.8 mg kg^−1^ h^−1^, with adjustments between 0.4 and 1.7 mg kg^−1^ h^−1^, the BIS value was maintained between 40 and 60, and blood pressure remained stable, highlighting the safety of ciprofol (Liang et al., 2023). In elderly patients, ciprofol also maintains stable hemodynamics, resulting in good postoperative recovery and lower CAM scores (Liu Z. et al., 2024). Notably, ciprofol is less toxic to the hepatic and renal systems, and patients with mild to moderate hepatic or renal impairment tolerate it well without the need for dosage adjustments (Hu et al., 2022; Liu S. B. et al., 2023). Its ability to maintain stable hemodynamics also ensure smooth kidney transplantation and therefore good post-transplantation renal function recovery (Qin et al., 2022).

### 4.2 Pain-free procedures

Ciprofol ensures a pain-free procedure. Studies have revealed that when used for gastrointestinal endoscopy, 0.2–0.5 mg kg^−1^ ciprofol has a 100% success rate (Teng et al., 2021). When used for colonoscopy, a dose of 0.4–0.5 mg kg^−1^ ciprofol provides a sedation effect similar to that of 2.0 mg kg^−1^ propofol, with a lower incidence of injection pain and no severe adverse events (Teng et al., 2021; Gao et al., 2024). Some studies revealed that the incidence of respiratory depression and hypotension is lower at doses of 0.2 mg kg^−1^ and 0.3 mg kg^−1^ than at 0.4 mg kg^−1^ ciprofol (Chen et al., 2023), although further research is needed to determine whether this dose provides the same efficacy and depth of anesthesia. For fiberoptic bronchoscopy, ciprofol at doses of 0.3 mg kg^−1^ and 0.4 mg kg^−1^ has successful anesthesia induction rates of 91.3% and 100%, respectively, with the option of administering one-third to one-quarter of the initial dose at 2-min intervals as needed and providing sedative effects comparable to those of propofol at doses of 1.2 mg kg^−1^ and 2.0 mg kg^−1^. Ciprofol also maintains more stable blood oxygen saturation and hemodynamics and is less likely to cause injection pain (Luo et al., 2022; Wu et al., 2022). After induction with 0.4 mg kg^−1^ ciprofol, maintaining anesthesia with a dose of 0.6–1.2 mg kg^−1^ h^−1^ during hysteroscopic examination provides success rates similar to those of 2.0 mg kg^−1^ propofol, with a significantly lower incidence of respiratory adverse events (4.0% vs 31.1%) (Lan et al., 2023). It is noteworthy that a Phase IIa trial found that a dose of 0.4 mg kg^−1^ of ciprofol had a higher incidence of adverse reactions related to muscle fasciculation compared to 2.0 mg kg^−1^ of propofol (4.5% vs 0%) (Gao et al., 2024). Subsequently, a Phase IIa study compared doses ranging from 0.2 to 0.5 mg kg^−1^ of ciprofol and found that muscle fasciculation occurred only in the 0.2 mg kg^−1^ group (6.8%), with muscle fasciculations not being dose-dependent (Gao et al., 2024). This may be attributed to the low doses of ciprofol used, leading to anesthesia-related seizures (Lu et al., 2023).

In summary, ciprofol is associated with more stable hemodynamics, a lower incidence of respiratory adverse events, safe anesthesia induction, a lower likelihood of injection pain, and greater patient acceptance than propofol is and ensures a pain-free procedure. However, since various pain-free diagnostic and therapeutic procedures involve different intensities of stimulation and varying demands on the respiratory system, the optimal doses of ciprofol required in different settings should be explored further to ensure that the desired depth of anesthesia can be achieved under the safest conditions.

### 4.3 Sedation in the ICU

ICU patients often experience severe pain, anxiety, and discomfort, and many require mechanical ventilation to maintain respiration, which increases the likelihood of agitation (Dallı Ö et al., 2023; Bauerschmidt et al., 2023). Therefore, prolonged sedation is often required in the ICU setting (Bauerschmidt et al., 2023). A phase I study involving healthy volunteers revealed that ciprofol effectively maintains sedation via continuous intravenous infusion without drug accumulation, and patients can tolerate it for at least 12 h. Furthermore, ciprofol is associated with milder respiratory depression and less circulatory impact than propofol is (Hu et al., 2021).

For sedation in the ICU, the recommended regimen involves an initial bolus dose of 0.1 mg kg^−1^ intravenously, followed by a maintenance dose of 0.3 mg kg^−1^ h^−1^, with the dose adjusted between 0.06 and 0.8 mg kg^-1^ h^−1^ to maintain a RASS sedation score of −2 to +1 (Liu et al., 2022; Liu Y. et al., 2023). This dosage regimen provides more stable hemodynamics and milder respiratory depression and is safe and well tolerated for at least 24 h (Liu Y. et al., 2023). Compared with propofol, ciprofol appears to be safer for prolonged infusion. Long-term infusion of propofol has been associated with hypertriglyceridemia (Pancholi et al., 2023), whereas continuous 24-h infusion of ciprofol does not lead to elevated serum triglyceride levels (Liu et al., 2022; Liu Y. et al., 2023). Additionally, prolonged infusion of propofol can result in propofol-related infusion syndrome (PRIS), with an incidence rate of 2.9% and a related mortality rate of up to 36.8% (Li W. K. et al., 2022). High dosage and prolonged use are recognized as major risk factors for the development of propofol infusion syndrome (Hemphill et al., 2019). Given that ciprofol is a more potent anesthetic, the cumulative dose during prolonged infusion is much lower than that of propofol, which theoretically may reduce the risk of PRIS. However, more clinical data are needed to further validate this hypothesis.

While the advantages of ciprofol over propofol are clear, research on its use for sedation in the ICU is still limited. Moreover, as a novel 2,6-disubstituted phenol derivative similar to propofol, the effects of long-term ciprofol infusion on adrenal cortical function remain unclear. Furthermore, there is currently no evidence to suggest that ciprofol improves long-term outcomes in ICU patients. More research is needed to clarify these aspects in the future.

## 5 Conclusion

Ciprofol, a new (GABA)-A receptor agonist, has a pharmacological mechanism similar to that of propofol. However, owing to the addition of a cyclopropyl group, ciprofol is a stronger anesthetic than propofol is, allowing for lower clinical doses. We believe that the lower clinical doses of ciprofol may be one of the key reasons for its association with lower incidences of hypotension, respiratory depression, and injection pain. This finding is consistent with the observation that lower doses of propofol tend to result in fewer adverse reactions. The primary difference in pharmacological effects between ciprofol and propofol may lie in anesthetic potency rather than adverse effects.

Overall, ciprofol has shown promising clinical outcomes and is suitable for use in both elderly and pediatric patients. Its performance in clinical settings thus far suggests excellent potential, although further exploration is needed to determine the optimal dose for various clinical scenarios.
